# Predicting protein subcellular locations using hierarchical ensemble of Bayesian classifiers based on Markov chains

**DOI:** 10.1186/1471-2105-7-298

**Published:** 2006-06-14

**Authors:** Alla Bulashevska, Roland Eils

**Affiliations:** 1 Theoretical Bioinformatics Department, German Cancer Research Center, Im Neuenheimer Feld 280, 69120 Heidelberg, Germany

## Abstract

**Background:**

The subcellular location of a protein is closely related to its function. It would be worthwhile to develop a method to predict the subcellular location for a given protein when only the amino acid sequence of the protein is known. Although many efforts have been made to predict subcellular location from sequence information only, there is the need for further research to improve the accuracy of prediction.

**Results:**

A novel method called HensBC is introduced to predict protein subcellular location. HensBC is a recursive algorithm which constructs a hierarchical ensemble of classifiers. The classifiers used are Bayesian classifiers based on Markov chain models. We tested our method on six various datasets; among them are Gram-negative bacteria dataset, data for discriminating outer membrane proteins and apoptosis proteins dataset. We observed that our method can predict the subcellular location with high accuracy. Another advantage of the proposed method is that it can improve the accuracy of the prediction of some classes with few sequences in training and is therefore useful for datasets with imbalanced distribution of classes.

**Conclusion:**

This study introduces an algorithm which uses only the primary sequence of a protein to predict its subcellular location. The proposed recursive scheme represents an interesting methodology for learning and combining classifiers. The method is computationally efficient and competitive with the previously reported approaches in terms of prediction accuracies as empirical results indicate. The code for the software is available upon request.

## Background

To cooperate towards the execution of a common physiological function (metabolic pathway, signal-transduction cascade, etc.), proteins must be localized in the same cellular compartment. There is an involved machinery within the cell for sorting newly synthesized proteins and sending them to their final locations. Identifying the destination of proteins in the cell is key to understanding their function. With the existence of many hypothetical proteins and proteins of unknown function, efforts to predict cellular localisation have been a vibrant field of research over the past few years. Efforts to identify extracellular proteins, for example, have been fueled by their possible use as therapeutic proteins.

A number of systems have been developed that support automated prediction of subcellular localization of proteins. Nakai and Kanehisa [1,2] developed an integrated expert system called PSORT to sort proteins into different compartments using sequentially applied "if-then" rules. The rules are based on different signal sequences, cleavage sites and the amino acid composition of individual proteins. At every node of an "if-then" tree a protein is classified into a category based on whether it satisfied a certain condition. Expert systems using production rules have a rich language for representing biological knowledge but are not well suited for reasoning under uncertainty. They are also unable to learn how to predict on its own and therefore very time consuming to update or adapt to new organisms.

Other computational prediction methods are based on amino acid composition using artificial neural nets (ANNs), such as NNSPL of Reinhardt and Hubbard [3], or support vector machines (SVMs), used in SubLoc [4].

TargetP of Emanuelsson et. al. [5] uses the existence of peptide signals, which are short subsequences of approximately 3 to 70 amino acids, to predict specific cell locations. For example, the KDEL, SKL and SV40-like motifs characterize endoplasmic reticulum (ER), peroxisome and nuclear proteins.

Horton and Nakai [6] used binary decision tree classifier and the Naive Bayes classifier to predict the subcellular location for the protein on the basis of an input vector of real valued feature variables calculated from the amino acid sequence. These features were mainly the outputs of different methods for detecting signal motifs and discriminant analysis on the amino acid content.

ProLoc introduced by Xie [7] can be classified as a method combining both amino acid composition and sorting signals. ProLoc searches also for compartment-specific domains.

As pointed out in [3], many genes are automatically assigned in large genome analysis projects, and these assignments are often unreliable for the 5'-regions. This can result in missing or only partially included leader sequences, thereby causing problems for sequence-motif-based localization algorithms. Additionally, proteins which are targeted to the extra-cellular space via non-classical secretory pathways do not possess N-terminal signal peptides.

Predictions based only on amino acid composition may lose some sequence order information. Chou in [8] proposed the so-called pseudo-amino-acid-composition. Trying to incorporate neighborhood information, Huang and Li [9] introduced a fuzzy *k*-NN method based on dipeptide composition. The count of occurrences of all 2-gram patterns is a 400 dimensional vector, which can be used to represent the protein sequence.

Yu et al. [10] proposed a predictive system called CELLO which uses SVMs based on n-peptide composition. The authors classified 20 amino acids into four groups (aromatic, charged, polar and nonpolar) to reduce the dimensionality of the input vector.

Bhasin and Raghava in [11] and Sarda et al. [12] used different physicochemical properties of amino acids, averaged over the entire protein or locally. However, such averaged values used as feature vectors for classification with SVMs do not utilize properly sequence order information.

Bickmore et al. [13] concluded that motifs and domains are often shared among proteins within the same sub-nuclear compartment. A novel concept of functional domain composition was introduced by Chou and Cai in [14].

LOCkey of Nair and Rost [15] conducts a similarity search on the sequence, extracts text from homologs and uses a classifier on the text features.

In this paper we propose a method, which uses the primary sequence of a protein for the prediction without employing methods for homology analysis, identification of sorting signals, searching for motifs and domains.

As a base learning algorithm our method uses Bayesian classification procedure. This scheme represents each class with a single probabilistic summary. In this paper we propose to use Markov chain model for the description of class density. Markov chains are broadly applied for analyzing biological sequence data (see e.g. Salzberg et.al. [16], Borodovsky et.al. [5], Lin et.al. [18]). Since in Markov chain model the probability of a symbol depends on the previous symbols, we believe that using Markov chains to model groups of sequences is the appropriate way to incorporate sequence order information. For the prediction of protein subcellular locations Markov chains were first used by Yuan [19].

To solve complex classification problems one can use hierarchical architectures, just like linear networks have led to multi-layer perceptrons. We exploit the idea of recursive partitioning, which was widely used in the classification with decision trees. We introduce a recursive algorithm called HensBC which constructs a hierarchical ensemble of Markov chains based Bayesian classifiers. A hierarchical system called LOCtree combining support vector machines and other prediction methods for predicting the subcellular compartment of a protein was introduced by Nair and Rost [20]. In contrast to our approach, the structure of the system is predefined by mimicking the mechanism of cellular sorting and using the evolutionary similarities among subcellular locations rather than learned from the data. The problem with this predefined architecture is that a prediction mistake at a top node could not be corrected at nodes lower in the hierarchy.

Our approach was also motivated by the idea that ensembles are often much more accurate than the individual classifiers. Ensemble methods have gained increasing attention over the past years, from simple averaging of individually trained neural networks over the combination of thousands of decision trees to *Random Forests *to the *boosting *of weak classifiers. AdaBoost (Adaptive Boosting) applied to probabilistic neural networks was used for protein subcellular localization based on amino acid composition in the paper of Guo et. al. [21].

Our algorithm presents an interesting methodology how the classifiers, learned on different portions of data, can be combined into a powerful system.

## Results and discussion

In order to demonstrate the encompassing applicability of our novel algorithmic approach to predict subcellular localization, we implemented our algorithm and tested it on disparate sequence datasets, including datasets of eukaryotic and prokaryotic sequences of Reinhardt and Hubbard, dataset constructed by Huang and Li, Gram-negative bacteria dataset, sequences of outer membrane and globular proteins, dataset of apoptosis proteins. In this section we will present and compare the overall predictive accuracies and the predictive accuracies for subcellular locations obtained with both procedures – single Markov chains based Bayesian classifier (BC) and hierarchical ensemble of this classifiers (HensBC) – for all datasets used in this study. Confusion matrices constructed according to the results of HensBC algorithm are provided [see Additional file 1]. We will also compare the results of HensBC with the results of previously published algorithms.

Table 1 is designed to compare the overall accuracies achieved with two approaches – BC and HensBC – on all datasets. One can observe from Table 1 that hierarchical ensemble can further improve the overall accuracy of the predictions of the single classifier. As will be shown and discussed further below, the predictive accuracies for distinct subcellular locations were improved dramatically.

Tables 2 and 3 show the results obtained for *eukaryotic and prokaryotic data*. Compared to BC, HensBC has managed to improve significantly the predictive accuracies for Extracellular and Nuclear locations for Data_Euk. The overall prediction accuracy of 78.7% achieved with HensBC for eukaryotic proteins is better than 73.0% achieved by Yuan [19] with fourth-order Markov chains, is comparable with 79.4% achieved by Hua and Sun [4] with SVMs and is lower than 85.2% of Huang and Li [9]. The overall result of 89.3% for prokaryotic proteins is slightly better than 89.1% of [19] achieved with fourth-order Markov chains.

For the results of experiments with *Dataset of Huang and Li *see Table 4. It is interesting, that for this dataset the HensBC was 17.3% superior than single Bayesian classifier. The predictive accuracies for Vacuole, Cytoplasm, Chloroplast, Peroxisome and Nuclear subcellular locations were improved dramatically. The overall prediction accuracy of 80.2% is actually the same (80.1%) achieved by Huang and Li [9] with fuzzy *k*-NN method. The only two classes, for which fuzzy *k*-NN method is superior than HensBC, are Extracellular and Chloroplast. Noticeably, the prediction performance for the smaller sized classes such as Cytoskeleton, Golgi and Vacuole achieved with HensBC is better than that of [9]. This implies that our approach gives better chance for the sequences of small classes in such a big dataset.

The confusion matrix for Data_SWISS [see Table 3 in Additional file 1] shows that the big part of the misclassification error results from confusing cytoplasmic proteins with nuclear proteins and vice versa. The same fact can be stated for Data_Euk [see Table 1 in Additional file 1] and was also observed by Horton and Nakai [6] for yeast data. Confusion of cytoplasmic and mitochondrial proteins is also observed for Data_SWISS and Data_Euk. This fact was also noted by Guo et al. [21]. All these confusions could possibly be considered as an illustration of the idea that some compartments are more similar to each other than others. In the hierarchical system LOCtree of Nair and Rost [20], Cytosol and Mitochondria locations are grouped together into "intermediate" location class Cytoplasm, and Cytoplasm together with Nucleus build "intermediate" location Intracellular separated from secretory pathway proteins. The confusion matrix for Data_SWISS was encouraging in that only a small fraction of extracellular proteins were predicted as proteins sorted to the organelles, e.g. belonging to either of the following locations: endoplasmic reticulum (ER), Golgi apparatus, peroxisome, lysosome or vacuole.

Since we wanted to compare our results for *Gram-negative bacteria dataset *with previously published, we followed the method of Gardy et. al. [22] in evaluating classifier for proteins resident at dual localization sites. For the sequences with dual locations, if one of their locations is predicted, we will consider them as correctly predicted. The results of the experiments using this evaluation method are reported in Table 5. The overall prediction accuracy of our method reaches 83.2%, which is 8.4% higher than that of PSORT-B (74.8%) introduced by Gardy et. al. [22], though we do not use specialized algorithms or particular input vectors for each localization site. Compared with PSORT-B, our method gives significantly better predictive performances for all the localization sites except outer membrane proteins (OMPs). PSORT-B reaches 90.3% for OMPs, however, it utilizes an extra module based on identification of frequent sequences occurring only in beta-barrel proteins. We reach the accuracy of 87.1% for outer membrane proteins, which is also very high. The identification of OMPs is of particular interest, because they are on the surface of the bacteria and so are the most accessible targets to develop new drugs against. These surface-exposed proteins are also useful for diagnosing diseases as a means to detect the bacteria.

It is remarkable, that for periplasmic proteins our method reaches the accuracy of 79.1%, which is 21.5% higher than that of PSORT-B.

However, the predictive accuracy of our method is 5.7% lower than that of CELLO, reported by Yu et. al. [10], and 6.6% lower than that of P-CLASSIFIER introduced by Wang et. al. [23].

We tried also to consider dual localization categories as separate locations as it is done in the work of Horton et. al. [24]. The problem therefore becomes a classification task with 9 classes. The prediction results achieved with HensBC are reported in confusion matrix of Table 5 in Additional file 1. From the confusion matrix we see that five major locations are good predicted and only a small number of proteins from these locations are misclassified as dually localized. Many of the proteins labelled as "Cytoplasmic/Inner membrane" are misclassified as either "Cytoplasmic" or "Inner membrane"; labelled as "Inner Membrane/Periplasmic" are misclassified as either "Inner Membrane" or "Periplasmic", labelled as "Outer membrane/Extracellular" are misclassified as either "Outer membrane" or "Extracellular". Like in [24], we gave partial credit for partially correct predictions as shown in Table 6. With this evaluation method we achieved the overall accuracy of 80.4%.

The results of *discriminating outer membrane from globular proteins *are shown in Table 7. For this binary classification problem we achieved with HensBC the sensitivity (recall) of 94.2% and specificity (precision) of 89.6%, which is higher than 84% and 78% reported by Gromiha and Suwa [25]. The more recent work of Park et. al. [26] attains the overall accuracy of 93.9% on a redundancy reduced (sequences with a high degree of similarity to other sequences were removed) dataset of Gromiha and Suwa [25]. The sensitivity of OMP prediction achieved in this work is 90.9%.

Table 8 presents the results with *Apoptosis proteins*. Even with the single Markov chains based Bayesian classifier we reached the overall accuracy of 85.7%, which is 13.2% higher than reached by Zhou and Doctor [27] with covariant discriminant algorithm. The HensBC approach reaches the overall accuracy of 89.8%.

## Conclusion

Automated prediction of protein subcellular localization is an important tool for genome annotation and drug discovery. Here we report the subcellular location prediction method, which make use of the primary sequence information. The method constructs in a greedy top-down fashion hierarchical ensembles consisting of gating Markov chain based Bayesian classifiers, which gate the protein in question either to the leaf labelled with the output, or to the next classifier.

The employment of Markov chain models for the description of class conditional distributions allows to make better use of sequence order and contextual information. The final ensemble can contain multiple probabilistic summaries for each location class, which provide the opportunity of better representing more diverse location classes.

The method can be effectively implemented and is computationally efficient. It demonstrates good results in the empirical evaluation and is competitive with previously reported approaches in terms of prediction accuracies. It outperforms the system PSORT-B for Gram-negative bacteria data, improves significantly previously obtained results for the apoptosis proteins and for discriminating outer membrane and globular proteins.

We believe that our method offer an interesting way of creating well-performing classifiers for very large datasets with skewed class distributions.

Because it does not utilize specialized algorithms or particular inputs for localization classes, it can be used for different organisms.

Some improvements over the proposed approach are possible. In particular, the application of post-pruning can be investigated.

One possible venue for future research may be to use Bayesian classifiers based on variable memory Markov models (VMMs) [28].

Because the method we propose in this paper need only raw sequence data and can be applied without assuming any preliminary biological information, it bears the advantage of being applicable to various classification tasks in multiple areas of biological analysis. The method could be potentially useful for classification of G-protein coupled receptors (GPCRs), nuclear receptors, enzyme families, analysis of proteins function and prediction of RNA binding proteins. Our method might become a powerful tool for the analysis of huge amount of sequence data.

The idea of refining the existing classification schemes using multi-level ensembles should be investigated in other applications or integrated with other classification methods in order to verify whether it is generally applicable.

## Methods

### Datasets

We applied our method to the previously published datasets.

#### Dataset of Reinhardt and Hubbard (Data_Euk and Data_Prok)

This dataset developed by Reinhardt and Hubbard [3] has been widely used for subcellular location studies, e.g. by Yuan [19], Hua and Sun [4] and Sarda et. al. [12]. It included only globular proteins. As shown in Tables 9 and 10, there are 2427 protein sequences from eukaryotic species classified into four location groups (Data_Euk) and 997 prokaryotic sequences, which were assigned to three location categories (Data_Prok).

#### Dataset of Huang and Li (Data_SWISS)

The second dataset was constructed by Huang and Li [9]. Sequences were selected from all eukaryotic proteins with annotated subcellular location in SWISS-PROT release 41.0 [29]. All proteins with ambiguous words such as PROBABLE, POTENTIAL, POSSIBLE and BY SIMILARITY and also proteins with multiple annotations of locations were excluded. The transmembrane proteins were excluded also. The number of proteins and their distributions in 11 categories are listed in Table 11.

#### Gram-negative bacteria dataset (Data_Gram)

Research of disease-causing, or pathogenic, bacteria, including the organisms responsible for food poisoning, water-borne diseases and meningitis, is of great importance.

While many bacteria have only 3 primary localization sites, Gram-negative bacteria have 5 major subcellular localization sites.

A manually curated dataset of proteins of experimentally known subcellular localization was constructed by Gardy et. al. [22]. For our experiments we used the newest version of the data set (see Table 12). The dataset comprises 1444 proteins resident at a single localization site and 147 proteins resident at dual localization sites.

#### Dataset of outer membrane and globular proteins (Data_OMP)

β-barrel membrane proteins (outer membrane proteins or OMPs) are found in the outer membranes of bacteria, mitochondria and chloroplast. They differ from the all β-structural class of globular proteins and have different structural motifs compared with α-helical membrane proteins.

We tested our approach on a dataset of 377 annotated OMPs and 674 globular proteins belonging to all structural classes. This dataset was constructed by Gromiha and Suwa [25].

#### Apoptosis proteins (Data_Apoptosis)

Apoptosis, or programmed cell death, is currently an area of intense investigation. To understand the apoptosis mechanism and functions of various apoptosis proteins, it would be helpful to obtain information about their subcellular location.

Zhou and Doctor [27] constructed a training dataset, containing 98 apoptosis proteins classified into four categories (see Table 13).

### Markov chain models

Markov models are the probabilistic models for sequences of symbols. Markov chain is a model that generates sequences in which the probability of a symbol depends on the previous symbols. We used in this study the *first-order Markov chain model*.

Let Σ be the alphabet of the twenty amino acids of which protein sequences are composed. Let *s *be a protein sequence of length *n*,

*s *= σ_1_*σ*_2 _... *σ*_*i*-1_*σ*_*i *_... *σ*_*n*_,

where σ_*i *_∈ Σ is the amino acid residue at sequence position *i*. For a first-order Markov model the frequencies of the residues in position *i *depend on the residue in position *i *- 1. The probability of a sequence *s *to be generated from the model *c *is given by the Markov chain formula:

Pc(s)=Pc(σ1)∏i=2nPc(σi|σi−1)
MathType@MTEF@5@5@+=feaafiart1ev1aaatCvAUfKttLearuWrP9MDH5MBPbIqV92AaeXatLxBI9gBaebbnrfifHhDYfgasaacH8akY=wiFfYdH8Gipec8Eeeu0xXdbba9frFj0=OqFfea0dXdd9vqai=hGuQ8kuc9pgc9s8qqaq=dirpe0xb9q8qiLsFr0=vr0=vr0dc8meaabaqaciaacaGaaeqabaqabeGadaaakeaacqWGqbaudaahaaWcbeqaaiabdogaJbaakiabcIcaOiabdohaZjabcMcaPiabg2da9iabdcfaqnaaCaaaleqabaGaem4yamgaaOGaeiikaGccciGae83Wdm3aaSbaaSqaaiabigdaXaqabaGccqGGPaqkdaqeWaqaaiabdcfaqnaaCaaaleqabaGaem4yamgaaOGaeiikaGIae83Wdm3aaSbaaSqaaiabdMgaPbqabaGccqGG8baFcqWFdpWCdaWgaaWcbaGaemyAaKMaeyOeI0IaeGymaedabeaakiabcMcaPaWcbaGaemyAaKMaeyypa0JaeGOmaidabaGaemOBa4ganiabg+Givdaaaa@4FD8@

Here *P*^*c*^(*σ*_1_) is the probability of observing amino acid *σ*_1 _in the first position of the sequence and *P*^*c*^(σ_*i*_|*σ*_*i*-1_) is the conditional probability of observing residue *σ*_*i *_in position *i*, given that *σ*_*i*-1 _is in position *i *- 1. The probability *p*^*c*^(*σ*) of observing symbol *σ *∈ Σ in the first position of the sequences generated from the model, the so-called *initial state probability*, and the conditional probability *P*^*c*^(*σ*|*σ*') of observing symbol *σ *right after *σ*', called *transition probability*, constitute the *parameters *of the Markov chain model. Given these parameters, the probability distribution over a set of sequences of different lengthes is completely specified.

The parameters of the model *c *are typically *estimated *from the set of trusted examples called *training set *– the set of sequences, which are assumed to be generated from the model *c*.

The initial state probability can be calculated as:

Pc(σ)=Fc(σ)∑σ′∈ΣFc(σ′)∀σ∈Σ
 MathType@MTEF@5@5@+=feaafiart1ev1aaatCvAUfKttLearuWrP9MDH5MBPbIqV92AaeXatLxBI9gBaebbnrfifHhDYfgasaacH8akY=wiFfYdH8Gipec8Eeeu0xXdbba9frFj0=OqFfea0dXdd9vqai=hGuQ8kuc9pgc9s8qqaq=dirpe0xb9q8qiLsFr0=vr0=vr0dc8meaabaqaciaacaGaaeqabaqabeGadaaakeaafaqabeqacaaabaGaemiuaa1aaWbaaSqabeaacqWGJbWyaaGccqGGOaakiiGacqWFdpWCcqGGPaqkcqGH9aqpdaWcaaqaaiabdAeagnaaCaaaleqabaGaem4yamgaaOGaeiikaGIae83WdmNaeiykaKcabaWaaabeaeaacqWGgbGrdaahaaWcbeqaaiabdogaJbaakiabcIcaOiqb=n8aZzaafaGaeiykaKcaleaacuWFdpWCgaqbaiabgIGiolabfo6atbqab0GaeyyeIuoaaaaakeaacqGHaiIicqWFdpWCcqGHiiIZcqqHJoWuaaaaaa@4C71@

where *F*^*c*^(*σ*) denote the occurring frequency of amino acid *σ *in the first positions of sequences from the training set.

The transition probability can be calculated as:

Pc(σ|σ′)=Fc(σ′,σ)∑σ″∈ΣFc(σ′,σ″)∀σ,σ′∈Σ
 MathType@MTEF@5@5@+=feaafiart1ev1aaatCvAUfKttLearuWrP9MDH5MBPbIqV92AaeXatLxBI9gBaebbnrfifHhDYfgasaacH8akY=wiFfYdH8Gipec8Eeeu0xXdbba9frFj0=OqFfea0dXdd9vqai=hGuQ8kuc9pgc9s8qqaq=dirpe0xb9q8qiLsFr0=vr0=vr0dc8meaabaqaciaacaGaaeqabaqabeGadaaakeaafaqabeqacaaabaGaemiuaa1aaWbaaSqabeaacqWGJbWyaaGccqGGOaakiiGacqWFdpWCcqGG8baFcuWFdpWCgaqbaiabcMcaPiabg2da9maalaaabaGaemOray0aaWbaaSqabeaacqWGJbWyaaGccqGGOaakcuWFdpWCgaqbaiabcYcaSiab=n8aZjabcMcaPaqaamaaqababaGaemOray0aaWbaaSqabeaacqWGJbWyaaaabaGaf83WdmNbayaacqGHiiIZcqqHJoWuaeqaniabggHiLdGccqGGOaakcuWFdpWCgaqbaiabcYcaSiqb=n8aZzaagaGaeiykaKcaaaqaaiabgcGiIiab=n8aZjabcYcaSiqb=n8aZzaafaaaaiabgIGiolabfo6atbaa@57A6@

where *F*^*c*^(*σ*', *σ*) denote the occurring frequency of amino acid pair (*σ*', *σ*) in the training set. The statistics of consecutive pair-residues will generate a matrix with 20 * 20 elements.

This way of estimating parameters of the model is called *maximum likelihood estimation*, because it can be shown that using the frequencies to calculate the probabilities maximises the total probability of training sequences given the model (the *likelihood*).

For a detailed description of Markov models we refer the reader to the book by Durbin et. al. [30].

### Bayesian classifiers based on Markov chains

In supervised machine learning a learning algorithm is given a training set including labeled instances. As a result of training an algorithm produces a classifier, which is later used to predict the unknown class or correct label for a new unlabeled instance.

A widely applied method in the machine learning and statistical community is Bayesian classification, which uses the Bayes' theorem (the Bayes rule). According to it the class for an unlabeled instance *s *should be the one which maximizes the posterior probability:

P(c|s)=P(c)P(s|c)P(s)=P(c)P(s|c)∑cP(c)P(s|c)
 MathType@MTEF@5@5@+=feaafiart1ev1aaatCvAUfKttLearuWrP9MDH5MBPbIqV92AaeXatLxBI9gBaebbnrfifHhDYfgasaacH8akY=wiFfYdH8Gipec8Eeeu0xXdbba9frFj0=OqFfea0dXdd9vqai=hGuQ8kuc9pgc9s8qqaq=dirpe0xb9q8qiLsFr0=vr0=vr0dc8meaabaqaciaacaGaaeqabaqabeGadaaakeaacqWGqbaucqGGOaakcqWGJbWycqGG8baFcqWGZbWCcqGGPaqkcqGH9aqpdaWcaaqaaiabdcfaqjabcIcaOiabdogaJjabcMcaPiabdcfaqjabcIcaOiabdohaZjabcYha8jabdogaJjabcMcaPaqaaiabdcfaqjabcIcaOiabdohaZjabcMcaPaaacqGH9aqpdaWcaaqaaiabdcfaqjabcIcaOiabdogaJjabcMcaPiabdcfaqjabcIcaOiabdohaZjabcYha8jabdogaJjabcMcaPaqaamaaqababaGaemiuaaLaeiikaGIaem4yamMaeiykaKIaemiuaaLaeiikaGIaem4CamNaeiiFaWNaem4yamMaeiykaKcaleaacqWGJbWyaeqaniabggHiLdaaaaaa@5F36@

Estimating the probability *P*(*s*) is unnecessary because it is the same for all classes. The remaining probabilities are estimated from the training set. Priors *P*(*c*) are estimated as the proportion of samples of the class *c*. The estimation of the class-conditional densities involves *K *subproblems (*K *is the number of classes), in which each of the density is estimated based on the data belonging to the class only. The core part of the design of the Bayesian classifier is the selection of the appropriate model for the generation of data instances of each class. In our application we assume that the sequences of each class are generated from a first-order Markov chain model. Thus after estimating the parameters of each class model, the term *P*(*s*|*c*) denoting the prior probability of a sequence *s *to belong to the class *c *can be computed using the formula for *P*^*c*^(*s*) from the previous section.

### Induction of hierarchical ensemble

After having explained the induction of Bayesian classifiers based on Markov chain models, we introduce now our algorithm, which constructs a hierarchical ensemble of such classifiers. The algorithm uses a *divide and conquer *strategy that attacks a complex problem by dividing it into simpler problems and recursively applies the same strategy to the subproblems.

In the learning phase, a tree-like hierarchy will be produced with classifiers at each non-terminal node. Starting with a tree containing only one node and the entire training set of sequences associated with this node, the following algorithm is applied:

Input: a set of protein sequences labelled with their location classes.

• If the majority of the sequences at the current node belong to a single class or if the size of the node (the number of the sequences at the node) is smaller than *ThreshSize*, create a leaf node labelled with the most represented class.

• Otherwise, learn classifier from the sequences at the current node and save this classifier in the node;

• create as many child nodes as there are classes at the node;

• apply the learned classifier on the sequences, e.g. predict for each sequence its class and assign this sequence to the corresponding child node;

• for each child node, call the algorithm recursively.

The base classification procedure (Bayesian classification based on Markov chains) repeatedly presents protein sequences at each node, producing a disjunctive partition of them. The intuition behind this procedure is that at some level of the hierarchy the majority of the sequences, which arrive at each child's node after the application of the classifier at the father's node, will have one class label, so that there will be no need to split the node. As "majority" is an ambiguous term, we should explain at this point how we implemented this stopping rule. Our algorithm stops splitting and creates a leaf node, if the fraction of the sequences at this node that do not belong to the most represented class do not exceed the *Thresh *= 0.01. If it is not the case, the base classification procedure is invoked again to further subdivide the data.

**Figure 1:**
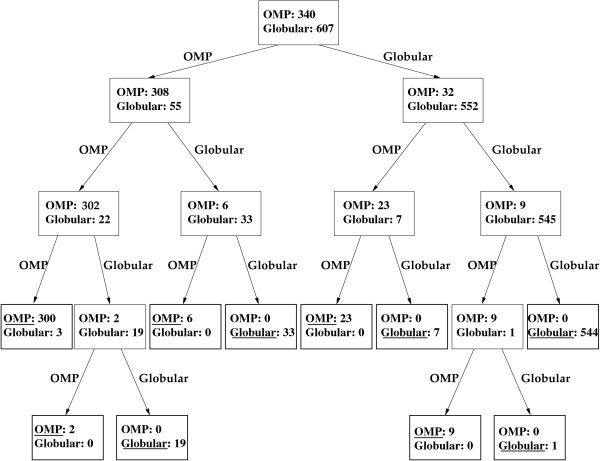
Hierarchical ensemble constructed for the task of discriminating Outer membrane (OMP) from Globular proteins. Each node is labelled with the numbers of OMPs and Globular proteins associated with it. At each internal node a Markov chains based Bayesian classifier is learned from the associated proteins, saved in the node and applied on these proteins. Two edges originate from each internal node, labelled "OMP" and "Globular", corresponding to the child nodes, which become proteins assigned by the classifier to OMP or Globular class, respectively. The final localization class to be outputed at each leaf node in the application phase is underlined.

The number of descendants of each node is equal to the number of classes that fall at this node, so the generated trees are in general not binary. Figure 1 shows schematically a hierarchical ensemble constructed for the task of discriminating Outer membrane (OMP) from Globular proteins.

Like any hierarchical induction algorithm, our algorithm can overfit the data by constructing an overly detailed tree. That is the reason why we adopt another stopping rule which uses the threshold for the size of the node *ThreshSize *as a kind of pre-pruning scheme.

In the application phase, the query protein sequence visits in turn the classifiers saved in the nonterminal nodes, which predict the new destination for it, till it arrives at the terminal node, labelled with the class to be outputed.

**Figure 2:**
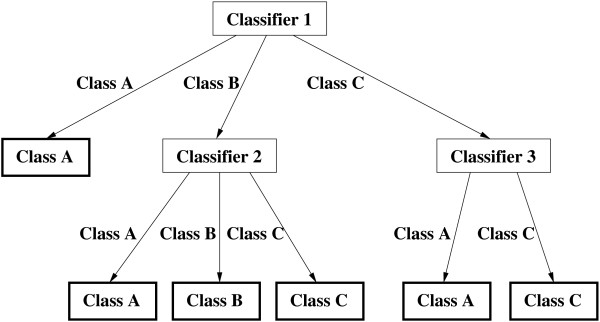
Hierarchical ensemble consisting of 3 Classifiers constructed to solve 3-class classification problem.

We would like to point out the important advantage of our hierarchical approach over the single Bayesian classification. Bayesian classification is a generative approach, which employs a set of generative models (Markov chain models in our case), each of which is trained over one class of data. For heterogeneous classes it could be problematic to represent such class with a single probabilistic summary. In our final hierarchical model, in contrast, each class can be represented not with single Markov chain model, but with multiple Markov chain models. Have a look at Figure 2, which depicts an architecture consisting of 3 Classifiers constructed to solve 3-class classification problem. In the hierarchical model corresponding to this architecture Class A is represented with 3 Markov chain models and Class C with 2 models. Another related point is that, while going down the hierarchy during the training phase, the subsets of sequences of each class will become smaller and not so diverse, so that they could be more effectively described by the Markov chain model. The ability to comprise multiple probabilistic summaries for each class should constitute the reason for an improvement over the accuracy of the single classifier.

We denote the constructed hierarchy as an *ensemble *of classifiers to emphasize the fact that not one classifier is used in the classification decision process, but multiple classifiers. An important factor influencing the accuracy of the ensemble is the *diversity *of base (or component) classifiers forming the ensemble. Each base classifier should work well on different parts of the given data set. If we have again a look at Figure 2, we can see that there are sequences of Class A, which Classifier 1 is not able to classify correctly into Class A, but Classifier 2 and Classifier 3 are. So the classifiers forming this hierarchical ensemble are diverse or heterogeneous. Our method presents a framework how not only to learn diverse classifiers, but to combine them so, that they will actually work well together in a composite classifier. For example, the sequences of Class A, which Classifier 1 is not able to classify correctly, should be delivered to the Classifiers 2 and 3. For these sequences the Classifier 1 plays this gating function.

It is interesting to relate our method to the known ensemble methods such as *bagging *(bootstrap aggregating) of Breiman [31] and *boosting *of Freund and Schapire [32], which rely on learning a set of diverse base classifiers typically by using different subsamples of the training set.

The work process of a boosting algorithm is to repeatedly reweight the examples in the training set and rerun the base learning algorithm to concentrate on the hardest examples. AdaBoost is an adaptive algorithm to boost a sequence of classifiers, in which the weights are updated dynamically according to the errors in previous learning. In contrast to boosting, which employs the *combination approach *during the application phase, where the final outcome is composed using the predictions of all base classifiers, our algorithm employs the *selection approach*, where the final class predictions are produced by one of the classifiers of the ensemble and other classifiers are dynamically selected to gate the query instance to this final classifier.

Our method is also related with the novel approach of *delegation*, which was introduced by Ferri et. al. [33] and can be summarized by the motto: let others do the things that you cannot do well. Delegation is a serial (not parallel or hierarchical) multiclassifier method, which constructs ensembles of *cautious classifiers*. Cautious classifier, introduced by Ferri [34], classifies only the examples for which it is sure of being able to make the right decision, and abstains for the rest of its inputs, leaving them for another classifier. Since each classifier retains part of the examples, the next classifier has fewer examples for training and, hence, the classification process can be much more efficient. The resulting classifier is not a hierarchy of classifiers, but a decision list.

We should also mention the relationship of our method to the *recursive naive Bayes *presented by Langley [35], which uses the naive Bayesian classifier as the base learning algorithm and was applied to domains with nominal attributes. The author claims that this recursive approach should outperform the simple Naive Bayes for the cases which occupy noncontiguous regions of the instance space, because one cannot represent such disjunctive situations with a single probabilistic summary for each class. The superiority of recursive naive Bayes over single naive Bayes was shown on synthetic data specifically generated.

### Validation

To compare the prediction performance of the classification methods we used standard performance measures.

The Bayesian classification approach was validated with *Jack-knife test *(or leave-one-out cross-validation) [36]. By *Jack-knife test *the learning step is performed with all sequences except the one, for which the location is to be predicted. The prediction quality was evaluated by the overall prediction accuracy and prediction accuracy for each location:

overall accuracy=∑c=1KT(c)N       accuracy(c)=T(c)N(c),
 MathType@MTEF@5@5@+=feaafiart1ev1aaatCvAUfKttLearuWrP9MDH5MBPbIqV92AaeXatLxBI9gBaebbnrfifHhDYfgasaacH8akY=wiFfYdH8Gipec8Eeeu0xXdbba9frFj0=OqFfea0dXdd9vqai=hGuQ8kuc9pgc9s8qqaq=dirpe0xb9q8qiLsFr0=vr0=vr0dc8meaabaqaciaacaGaaeqabaqabeGadaaakqaabeqaaiabd+gaVjabdAha2jabdwgaLjabdkhaYjabdggaHjabdYgaSjabdYgaSjaaykW7cqWGHbqycqWGJbWycqWGJbWycqWG1bqDcqWGYbGCcqWGHbqycqWGJbWycqWG5bqEcqGH9aqpdaWcaaqaamaaqadabaGaemivaqLaeiikaGIaem4yamMaeiykaKcaleaacqWGJbWycqGH9aqpcqaIXaqmaeaacqWGlbWsa0GaeyyeIuoaaOqaaiabd6eaobaaaeaacaaMc8UaaGPaVlaaykW7caaMc8UaaGPaVlaaykW7caaMc8UaemyyaeMaem4yamMaem4yamMaemyDauNaemOCaiNaemyyaeMaem4yamMaemyEaKNaeiikaGIaem4yamMaeiykaKIaeyypa0ZaaSaaaeaacqWGubavcqGGOaakcqWGJbWycqGGPaqkaeaacqWGobGtcqGGOaakcqWGJbWycqGGPaqkaaGaeiilaWcaaaa@729C@

where *N *is the total number of sequences, *N*(*c*) is the number of sequences observed in location *c, K *is the number of locations and *T*(*c*) is the number of correctly predicted sequences of location *c*.

The results of HensBC method were validated with 10-fold cross-validation procedure. In *k-fold cross-validation *the dataset is partitioned randomly into *k *equally-sized partitions, and learning and evaluation is carried out *k *times, each time using one distinct partition as the testing set and the remaining *k *- 1 partitions as the training set.

We have calculated also the Matthew's correlation coefficients [37] between the observed and predicted locations over a dataset:

MCC(c)=p(c)n(c)−u(c)o(c)(p(c)+u(c))(p(c)+o(c))(n(c)+u(c))(n(c)+o(c))
 MathType@MTEF@5@5@+=feaafiart1ev1aaatCvAUfKttLearuWrP9MDH5MBPbIqV92AaeXatLxBI9gBaebbnrfifHhDYfgasaacH8akY=wiFfYdH8Gipec8Eeeu0xXdbba9frFj0=OqFfea0dXdd9vqai=hGuQ8kuc9pgc9s8qqaq=dirpe0xb9q8qiLsFr0=vr0=vr0dc8meaabaqaciaacaGaaeqabaqabeGadaaakeaacqWGnbqtcqWGdbWqcqWGdbWqcqGGOaakcqWGJbWycqGGPaqkcqGH9aqpdaWcaaqaaiabdchaWjabcIcaOiabdogaJjabcMcaPiabd6gaUjabcIcaOiabdogaJjabcMcaPiabgkHiTiabdwha1jabcIcaOiabdogaJjabcMcaPiabd+gaVjabcIcaOiabdogaJjabcMcaPaqaamaakaaabaGaeiikaGIaemiCaaNaeiikaGIaem4yamMaeiykaKIaey4kaSIaemyDauNaeiikaGIaem4yamMaeiykaKIaeiykaKIaeiikaGIaemiCaaNaeiikaGIaem4yamMaeiykaKIaey4kaSIaem4Ba8MaeiikaGIaem4yamMaeiykaKIaeiykaKIaeiikaGIaemOBa4MaeiikaGIaem4yamMaeiykaKIaey4kaSIaemyDauNaeiikaGIaem4yamMaeiykaKIaeiykaKIaeiikaGIaemOBa4MaeiikaGIaem4yamMaeiykaKIaey4kaSIaem4Ba8MaeiikaGIaem4yamMaeiykaKIaeiykaKcaleqaaaaaaaa@7460@

Here, *p*(*c*) is the number of properly predicted proteins of location *c, n*(*c*) is the number of properly predicted proteins not of location c, *u*(*c*) is the number of under-estimated and *o*(*c*) is the number of over-estimated proteins.

## Authors' contributions

AB developed the method, designed and implemented the system. RE continiously supported the work and provided valuable comments. AB drafted the manuscript. All authors read and approved the final manuscript.

## Supplementary Material

Additional File 1 This file contains 7 Tables depicting the confusion matrices of prediction results achieved with HensBC for all data sets used in the study. Click here for file

## Figures and Tables

**Table 1:** Performance comparison of single Bayesian classifier (BC) and hierarchical ensemble of Bayesian classifiers (HensBC).

Dataset	BC-approach Accuracy (%)	HensBC-approach Accuracy (%)
Data_Euk	70.8	78.7
Data_Prok	89.0	89.3
Data_SWISS	62.9	80.2
Data_Gram	77.2	83.2
Data_OMP	89.9	91.2
Data_Apoptosis	85.7	89.8

**Table 2:** The predictive accuracy for subcellular locations of single Bayesian classifier (BC) and hierarchical ensemble of Bayesian classifiers (HensBC) for Data_Euk.

	BC-approach		HensBC-approach	
Cellular location	Accuracy (%)	MCC	Accuracy (%)	MCC
Cytoplasmic	78.8	0.53	76.3	0.63
Extracellular	61.5	0.59	78.8	0.76
Mitochondrial	52.3	0.41	53.0	0.53
Nuclear	74.0	0.64	87.7	0.73
Overall accuracy	**70.8**	-	**78.7**	-

**Table 3:** The predictive accuracy for subcellular locations of single Bayesian classifier (BC) and hierarchical ensemble of Bayesian classifiers (HensBC) for Data_Prok.

	BC-approach		HensBC-approach	
Cellular location	Accuracy (%)	MCC	Accuracy (%)	MCC
Cytoplasmic	95.1	0.81	95.5	0.82
Extracellular	74.8	0.75	72.9	0.75
Periplasmic	75.8	0.69	76.7	0.70
Overall accuracy	**89.0**	-	**89.3**	-

**Table 4:** The predictive accuracy for subcellular locations of single Bayesian classifier (BC) and hierarchical ensemble of Bayesian classifiers (HensBC) for Data_SWISS.

	BC-approach		HensBC-approach	
Cellular location	Accuracy (%)	MCC	Accuracy (%)	MCC
Chloroplast	53.6	0.53	75.8	0.74
Cytoplasm	52.7	0.38	75.7	0.67
Cytoskeleton	58.3	0.19	66.7	0.57
Endoplasmic ret	48.9	0.31	67.2	0.67
Extracellular	71.3	0.61	86.2	0.82
Golgi apparatus	11.8	0.07	20.6	0.23
Lysosome	78.6	0.52	80.2	0.69
Mitochondria	59.8	0.43	64.1	0.61
Nuclear	66	0.55	85.3	0.76
Peroxisome	38.5	0.33	58.2	0.57
Vacuole	24.1	0.23	51.9	0.57
Overall accuracy	**62.9**	-	**80.2**	-

**Table 5:** The predictive accuracy for subcellular locations of single Bayesian classifier (BC) and hierarchical ensemble of Bayesian classifiers (HensBC) for Data_Gram.

	BC-approach		HensBC-approach	
Cellular location	Accuracy (%)	MCC	Accuracy (%)	MCC
Cytoplasmic	84.6	0.67	76.9	0.71
Inner membrane	80.7	0.82	85.8	0.84
Periplasmic	75.9	0.66	79.1	0.71
Outer membrane	78.2	0.71	87.1	0.79
Extracellular	60.2	0.61	73.9	0.73
Overall accuracy	**77.2**	-	**83.2**	-

**Table 6:** Utility of predictions according to partial credit method. Label denotes true localization.

Label	Prediction	Utility
Cytoplasmic	Cytoplasmic	1
Cytoplasmic/Inner membrane	Cytoplasmic/Inner membrane	1
Cytoplasmic/Inner membrane	Cytoplasmic	0.5
Cytoplasmic	Cytoplasmic/Inner membrane	0.5
Cytoplasmic/Inner membrane	Inner Membrane/Periplasmic	0.333
Cytoplasmic	Inner membrane	0
Periplasmic	Cytoplasmic/Inner membrane	0
Periplasmic	Cytoplasmic	0

**Table 7:** The predictive accuracy for subcellular locations of single Bayesian classifier (BC) and hierarchical ensemble of Bayesian classifiers (HensBC) for Data_OMP.

	BC-approach		HensBC-approach	
Cellular location	Accuracy (%)	MCC	Accuracy (%)	MCC
OMP	90.7	0.79	94.2	0.82
Globular	89.5	0.79	89.6	0.82
Overall accuracy	**89.9**	-	**91.2**	-

**Table 8:** The predictive accuracy for subcellular locations of single Bayesian classifier (BC) and hierarchical ensemble of Bayesian classifiers (HensBC) for Data_Apoptosis.

	BC-approach		HensBC-approach	
Cellular location	Accuracy (%)	MCC	Accuracy (%)	MCC
Cytoplasmic	90.7	0.81	95.3	0.89
Plasma membrane	90	0.83	90	0.83
Mitochondrial	92.3	0.83	92.3	0.83
Other	50.0	0.57	66.7	0.80
Overall accuracy	**85.7**	-	**89.8**	-

**Table 9:** Eukaryotic sequences within each subcellular location group (Data_Euk).

Cellular location	Number of proteins
Cytoplasmic	684
Extracellular	325
Mitochondrial	321
Nuclear	1097
Sum	2427

**Table 10:** Prokaryotic sequences within each subcellular location group (Data_Prok).

Cellular location	Number of proteins
Cytoplasmic	688
Extracellular	107
Periplasmic	202
Sum	997

**Table 11:** Protein sequences within each subcellular location group (Data_SWISS).

Cellular location	Number of proteins
Chloroplast	1145
Cytoplasm	2465
Cytoskeleton	24
Endoplasmic	137
Extracellular	4228
Golgi	34
Lysosome	131
Mitochondria	1106
Nuclear	3419
Peroxisome	122
Vacuole	54
Sum	12865

**Table 12:** Bacterial proteins within each subcellular location group (Data_Gram).

Cellular location	Number of proteins
Cytoplasmic	278
Cytoplasmic/Inner membrane	16
Inner Membrane	309
Inner Membrane/Periplasmic	51
Periplasmic	276
Periplasmic/Outer membrane	2
Outer membrane	391
Outer membrane/Extracellular	78
Extracellular	190
Sum	1591

**Table 13:** Apoptosis proteins within each subcellular location group (Data_Apoptosis).

Cellular location	Number of proteins
Cytoplasmic	43
Plasma membrane	30
Mitochondrial	13
Other	12
Sum	98
