# Supplementary material for: Predicting protein subcellular locations using hierarchical ensemble of Bayesian classifiers based on Markov chains
Source: BMC Bioinformatics. 2006 Jun 14;7:298. doi: 10.1186/1471-2105-7-298 (PMC1525000; doi:10.1186/1471-2105-7-298)
Supplement: Additional File 1 — This file contains 7 Tables depicting the confusion matrices of prediction results achieved with HensBC for all data sets used in the study. [file 1471-2105-7-298-S1.pdf]

Additional material

Table 1
Confusion matrix of prediction results of HensBC approach for Data_Euk.

	Predicted group				
	Cytoplasmic	Extracellular	Mitochondrial	Nuclear	Sum
Cytoplasmic	**522**	30	46	86	684
Extracellular	28	**256**	9	32	325
Mitochondrial	87	14	**170**	50	321
Nuclear	84	18	33	**962**	1097
Sum	721	318	258	1130	2427

Table 2
Confusion matrix of prediction results of HensBC approach for Data_Prok.

	Predicted group			
	Cytoplasmic	Extracellular	Periplasmic	Sum
Cytoplasmic	**657**	1	30	688
Extracellular	8	**78**	21	107
Periplasmic	31	16	**155**	202
Sum	696	95	206	997

Table 3
Confusion matrix of prediction results of HensBC approach for Data_SWISS.

	Predicted group											
	Chlor	Cytop	Cytos	End	Ext	Gol	Lys	Mit	Nuc	Per	Vac	Sum
Chloroplast	**868**	96	0	5	31	1	2	85	50	7	0	1145
Cytoplasm	71	**1867**	7	12	97	4	9	131	253	13	1	2465
Cytoskeleton	0	3	**16**	0	0	0	0	1	4	0	0	24
Endoplasmic	4	11	0	**92**	12	0	3	5	8	1	1	137
Extracellular	48	170	0	10	**3643**	7	39	64	222	14	11	4228
Golgi	1	6	0	0	5	**7**	1	3	11	0	0	34
Lysosome	0	5	0	0	14	0	**105**	3	3	1	0	131
Mitochondria	82	145	0	5	44	1	5	**709**	102	12	1	1106
Nuclear	43	230	10	10	121	7	6	70	**2915**	4	3	3419
Peroxisome	6	20	0	1	5	0	1	13	5	**71**	0	122
Vacuole	0	2	0	1	12	0	2	3	6	0	**28**	54
Sum	1123	2555	33	136	3984	27	173	1087	3579	123	45	12865

Table 4
Confusion matrix of prediction results of HensBC approach for Data_Gram.

	Predicted group					
	Cytoplasm	Inner membrane	Periplasm	Outer membrane	Extracell	Sum
Cytoplasmic	**223**	13	37	14	3	290
Inner membrane	28	**291**	9	9	2	339
Periplasmic	23	11	**239**	18	11	302
Outer membrane	11	4	18	**378**	23	434
Extracellular	7	4	17	31	**167**	226
Sum	292	323	320	450	206	1591

Table 5

Confusion matrix of prediction results of HensBC approach for Data_Gram with 9 locations. Numbers in round brackets represent the number of correctly predicted proteins according to the partial credit method.

	Predicted group									
	Cyt	Cyt/In_m	In_m	In_m/Per	Per	Per/Out_m	Out_m	Out_m/Ext	Ext	Sum
Cyt	**213**	3	11	1	37	0	8	0	5	(214.5)/278
Cyt/In_m	10	**2**	3	0	1	0	0	0	0	(8.5)/16
In_m	21	1	**269**	2	9	0	4	0	3	(270.5)/309
In_m/Per	10	1	4	**22**	10	0	3	0	1	(29)/51
Per	19	3	7	5	**204**	0	19	1	18	(206.5)/276
Per/Out_m	0	0	0	0	1	**0**	1	0	0	(1)/2
Out_m	13	3	2	0	10	2	**335**	11	15	(341.5)/391
Out_m/Ext	0	0	0	0	1	0	11	**58**	8	(67.5)/78
Ext	7	0	4	1	14	0	18	11	**135**	(140.5)/190
Sum	293	13	300	31	287	2	399	81	185	(1279.5)/1591

Table 6

Confusion matrix of prediction results of HensBC approach for Data_OMP.

	Predicted group		
	OMP	Globular	Sum
OMP	**355**	22	377
Globular	70	**604**	674
Sum	425	626	1051

Table 7

Confusion matrix of prediction results of HensBC approach for Data_Apoptosis.

	Predicted group				
	Cytoplasmic	Plasma membrane	Mitochondrial	Other	Sum
Cytoplasmic	**41**	2	0	0	43
Plasma membrane	0	**27**	3	0	30
Mitochondrial	0	1	**12**	0	13
Other	3	1	0	**8**	12
Sum	44	31	15	8	98
